# Supplementary material for: Investigating the influence of physiologically relevant hydrostatic pressure on CHO cell batch culture
Source: Sci Rep. 2021 Jan 8;11:162. doi: 10.1038/s41598-020-80576-8 (PMC7794228; doi:10.1038/s41598-020-80576-8)
Supplement: Supplementary file 1 — Supplementary Information 1. [file 41598_2020_80576_MOESM1_ESM.zip › S1/IgG/Feb14 0 30 60 90/SS1 Feb10 2.pdf]

Data File C:\Users\P...mStation\1\Data\TK_CHO_IgG 2019-02-14 14-17-55\036-7-ML SS1 Feb10.D
Sample Name: ML SS1 Feb10

===

Acq. Operator : SYSTEM Seq. Line : 36
Sample Operator : SYSTEM
Acq. Instrument : HPLC-0039 Location : 7
Injection Date : 2/14/2019 4:49:18 PM Inj : 1
Inj Volume : 100.000 µl
Method : C:\Users\Public\Documents\ChemStation\1\Data\TK_CHO_IgG 2019-02-14 14-17-55 \CHO_IgG_LC.M (Sequence Method)
Last changed : 2/8/2019 3:17:23 PM by SYSTEM

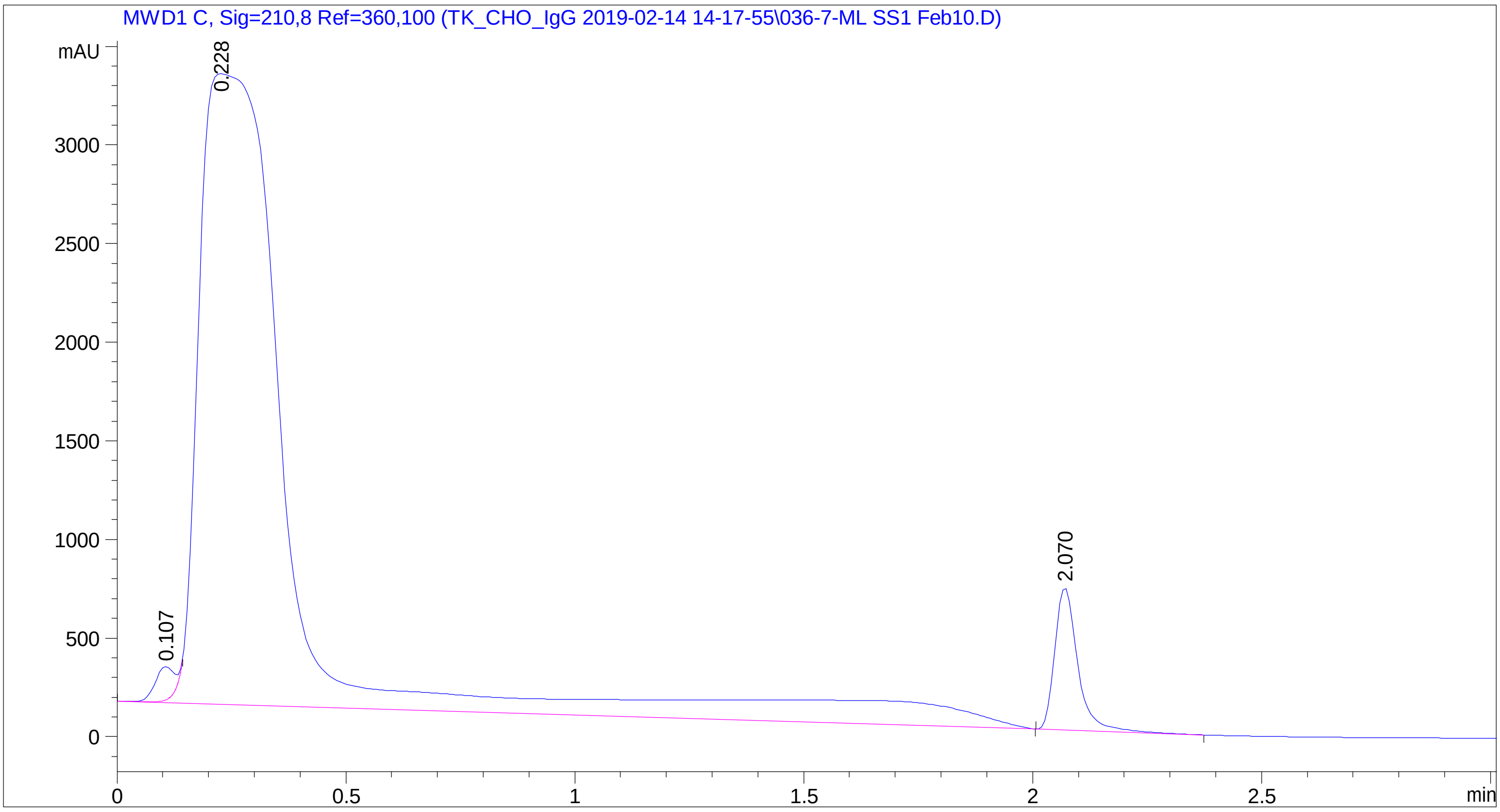

===

Area Percent Report

===

Sorted By : Signal
Multiplier : 1.0000
Dilution : 1.0000
Use Multiplier & Dilution Factor with ISTDs

Signal 1: MWD1 C, Sig=210,8 Ref=360,100

Peak #	RetTime [min]	Type	Width [min]	Area [mAU*s]	Height [mAU]	Area %
1	0.107	BV E	0.0450	479.79810	169.62402	1.0177
2	0.228	VB R	0.1831	4.41307e4	3196.23779	93.6071
3	2.070	BBA	0.0529	2534.12500	723.04761	5.3752

Totals : 4.71446e4 4088.90942

===

*** End of Report ***
